# THR-123, a novel BMP-7 mimetic that activates Akt phosphorylation and inhibits cardiomyocyte apoptosis and inflammation, protects the heart from myocardial injury in a Rat model

**DOI:** 10.3389/fcvm.2026.1729074

**Published:** 2026-06-25

**Authors:** Dattatreyamurty Bosukonda, Romesh R. Subramanian, Peter C. Keck, Philippe Bey, Frederic R. Carlson, William D. Carlson

**Affiliations:** 1 Division of Cardiology, Mass General Hospital, Boston, MA, United States; 2 Therapeutics by Design, Weston, MA, United States; 3 Thrasos Therapeutics, Hopkinton, MA, United States; 4 Harvard Medical School, Boston, MA, United States

**Keywords:** animal model, BMP mimetic, ischemia-Reperfusion (I/R) injury, myocardial infarction, THR-123

## Abstract

Acute myocardial infarction (AMI) continues to be the most common cause of heart failure despite the advancements in the treatment of Myocardial Infarction (MI) over the past 20 years. We have developed “BMP mimetics” that selectively activate the BMP signaling pathway, and do not induce bone formation. A BMP-7 mimetic, THR-123, is anti-inflammatory, anti-apoptotic, anti-fibrotic and promotes tissue regeneration. In an animal model of ischemia-reperfusion using LAD coronary occlusion-induced myocardial injury, THR-123 markedly decreased myocardial infarct size (84%) and pericardial inflammation. The mechanism of action of THR-123 was examined in three different cellular (cardiomyocytes) models. Mechanistically, THR-123 activates Akt phosphorylation and inhibits inflammation and apoptosis in cardiomyocytes. These results show that the BMP-7 mimetic (THR-123) protects cardiomyocytes, and limits infarct size after myocardial ischemia and reperfusion injury. THR-123 may provide a novel pharmacological intervention in myocardial injury.

## 1 Introduction

Ischemia-Reperfusion Injury following Acute Myocardial Infarction (AMI) is a leading cause of morbidity and mortality. There is a well-established clinical correlation between the size of a myocardial infarction and mortality in patients (1, 2). In the USA alone, acute myocardial infarction accounts for more than 1 million deaths annually (3). Myocardial infarction, colloquially known as “heart attack,” is caused by decreased or complete cessation of blood flow to a portion of the myocardium. Most myocardial infarctions are due to underlying coronary artery disease. The occlusion of one or multiple large epicardial coronary arteries for more than 20 to 40 min can lead to acute myocardial infarction. The occlusion leads to a lack of oxygen in the myocardium, which results in sarcolemmal disruption and myofibril relaxation (4). These changes are one of the first ultrastructural changes in the process of MI, which are followed by mitochondrial alterations. The prolonged ischemia ultimately results in liquefactive necrosis of myocardial tissue. The necrosis spreads from sub-endocardium to sub-epicardium. The sub-epicardium is believed to have increased collateral circulation, which delays its death (4). Depending on the territory affected by the infarction, the cardiac function can be compromised. Following myocardial infarction up to one billion cardiomyocytes become necrotic; this massive sudden loss of cardiomyocytes overwhelms any existing regenerative reserve. Due to the negligible regeneration capacity of the myocardium, the infarcted area heals by scar formation, and often, the heart remodeling is characterized by dilation, segmental hypertrophy of remaining viable tissue, and ultimately cardiac failure and death (5).

**Figure 1:**
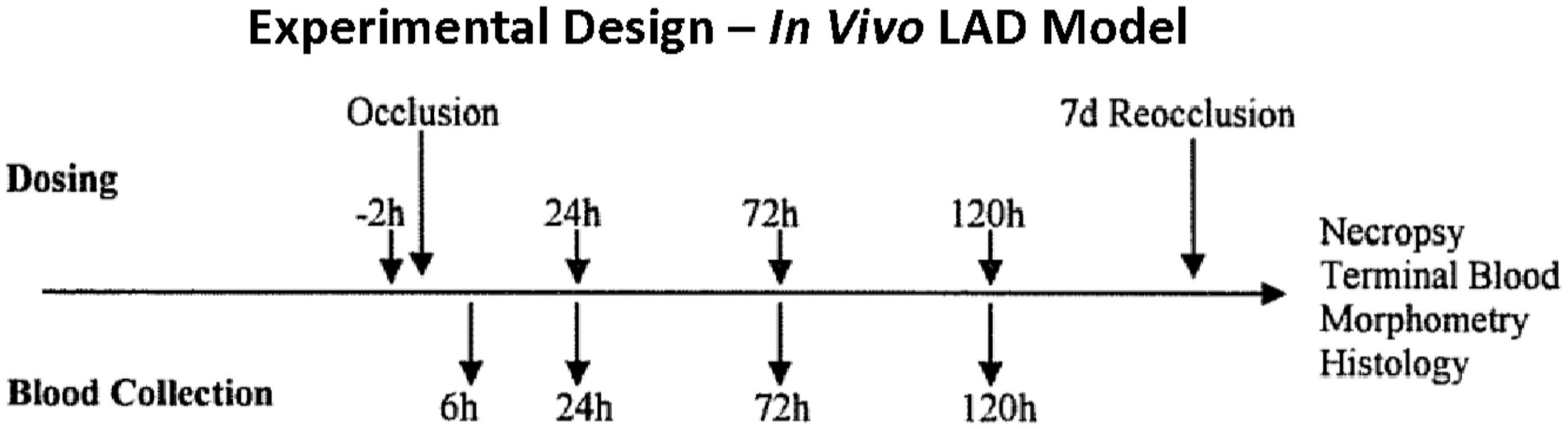
Animal study design, compound treatment and blood collection schedule in the animal model of myocardial injury following ischemia (LAD model).

Several therapies have been investigated for the treatment of ischemia-reperfusion and advanced decompensated heart failure (6). Most of these therapies targeted pathways downstream in the pathologic processes. As such, they affect only a single arm of a multifaceted process and are therefore limited in their beneficial effects. By looking upstream to targets that have broader effects, we have discovered agents that are safe, block the multifaceted cardiac cellular injury and are void of any deleterious effects (7). Our target is a developmental growth factor, bone morphogenic protein (BMP-7) that belongs to the TGF-*β* superfamily. BMP-7 signaling exerts important actions: blocking apoptosis, inflammation and fibrosis. BMP signaling also has significant effects on fibroblasts, cardiomyocytes and macrophages. Anti-fibrotic effects of BMP-7 have been reported in many systems and may be mediated, at least in part, through suppression of collagen synthesis by cardiac fibroblasts (8, 9). In cardiomyocytes, BMP-7/signaling attenuates hypertrophy by inhibiting TGF-*β* responses (8). In macrophages, BMP-7 has been reported to promote M2 polarization (10). Considering the absence of endogenous BMP-7 induction in infarcted and remodeling hearts, administration of exogenous BMP-7 has been suggested as a potential strategy to attenuate cardiac infarction and adverse remodeling.

BMP-7 signaling begins with the formation of a receptor complex that involves a dimer of BMPR-2 (type 2) receptors and a dimer of BMP type 1 receptors (BMPR-1A, ALK3; BMPR-1B, ALK6; and/or ACVR-1, ALK2) independently interacting with the BMP-7 dimer. This complex activates the canonical pathway (Smad signaling) and several non-canonical pathways such as the MAPK pathway, the PI3K/Akt pathway, the TAK1 pathway, and the Nf-KB pathway. The degree to which each of these is activated appears to be cell type dependent. In the case of cardiomyocytes, the PI3K/Akt pathway is predominant.

BMP-7 binds BMPR-IA(ALK3), BMPR-IB(ALK6), or the activin A receptor type 1 ACVR-I (ALK2). BMP activates both canonical and non-canonical pathways. In the canonical pathway, it activates BMPR-II which then activates BMP type 1 receptors to form the oligomeric receptor complex. This leads to phosphorylation of Smad-1/5/8 (a.k.a. Smad-1/5/9 or Smad-1/5/(8)9), which then complexes with Smad-4 and transmits the signal. In the non-canonical pathway involving XIAP, TAK1, and TAB1, the receptor complex is typically the first to be activated upon ligand binding, and then it triggers the downstream activation of theTAB1-TAK1complex, CIAP plays a role in modulating this activation by interacting with TAB1, not being directly activated by the receptor itself (11, 12). On the one hand, BMPR-IA (BMP type 1 receptor A, ALK3) was the most abundantly expressed receptor in the healthy LV myocardium in humans and mice. On the other hand, ACVR1 (Activin type I receptor, ALK2) was scarcely expressed, and BMPR-IB (BMP type 1 receptor B, ALK6) was virtually absent. The expression of Smad proteins in infarcted or fibrotic myocardium is different from that in the normal myocardium. No matter whether it is assessed in early or late-stage cardiomyopathy, the selective expression of Smad proteins is correlated with cardiac fibrosis and elevated collagen synthesis levels (13, 14). The expression of Smad-2, 3 and 4, which are activated by TGF-*β* is upregulated at the infarct scar as well as in the peri-ischemic border zone (14). Moreover, TGF-*β* may play an important role in modulating fibroblast phenotype and gene expression (13), promoting extracellular matrix deposition in the infarct by upregulating collagen and fibronectin synthesis and by decreasing matrix degradation through induction of protease inhibitors (15). Finding agents that block TGF-*β* induced deleterious effects or possibly reverse these processes at a checkpoint, is a subject of intense research. Recently Vukicevic et al. (16) have shown that the anti-BMP1.3 monoclonal antibody inhibited the TGF-*β* pathway, thus reducing myofibroblast activation to prevent cardiomyocyte apoptosis, reduce collagen deposition and preserve cardiac function after ischemia in a rodent model.

Evidence suggests that BMP-2, BMP-7, BMP-9, and BMP-10 can alleviate cardiac infarction and improve cardiac function after ischemia and reperfusion, primarily by reducing fibrosis, promoting cardiomyocyte survival, and enhancing cardiac repair (17). This suggests that they could be potential therapeutic targets for treating heart conditions like myocardial infarction (MI). Unfortunately, any potential therapeutic benefit of restoring BMP-7 functions using systemic rhBMP-7 is hampered by bioavailability, induction of ectopic bone formation, induction of neutralizing autoantibodies against BMPs, and a range of other potential adverse effects (18). To this end, we have designed small peptide agonists of BMP-7 based on the BMP-7 crystal structure (19). BMP-7 mimetic peptide THR-123 selectively binds the type I receptors ALK2 and ALK3, but it does not bind ALK6 (20). Unlike BMP-7, it does not have osteogenic activity and cannot induce ectopic bone formation (21). It exhibits robust anti-inflammatory, anti-apoptotic and anti-fibrotic, as well as regenerative, effects (20, 21).

Here, we evaluated THR-123 efficacy in alleviating myocardial injury caused by ischemia and reperfusion in the rat model of coronary artery ligation and studied the mechanism of action of THR-123 in three different *in vitro* cellular models. The non-canonical PI3K/Akt pathway is a major intracellular signaling pathway that regulates the survival, growth, and proliferation of cardiomyocytes (5). The *in vitro* data on neonatal rat cardiomyocytes presented below demonstrate that, following stimulation of inflammation with Adriamycin, treating the cells with THR-123 results in a dose dependent increase in Akt phosphorylation and concomitant decrease in cytokine IL-6 production.

## 2 Methods

### 2.1 THR-123

The starting points for the peptide mimetics considered herein were structural analogs of solvent accessible regions in the naturally occurring BMP-7 three-dimensional structure (21) identified as likely receptor binding sites. A detailed description of the design process has been published in Frontiers in Pharmacology (21).

THR-123 was prepared by standard solid-phase peptide synthesis on Fmoc-Ser(tBu) resin. The Chemical sequence of THR-123 is H-C*YFDDSSNVLC*KKYRS-OH (C*: Disulfide bridge). The starting amount of resin was 6 g with a substitution of 0.55 mmol/g (3.3 mmol). Side-chain protection of Fmoc amino-acid: tBu on Asp, Tyr, Ser; Trt on Asn, Cys: Boc on Lys; Pbf on Arg. Fmoc deprotection was achieved with 20% piperidine in DMF or 20% piperidine + 5% HOBt in DMF. Fmoc-amino acid coupling on to the resin was performed using the DIC and HOBt method. All couplings were completed in less than 2 h except for residue 11 Fmoc-Cys that required the addition of PyBOP to complete the reaction. The Kaiser test was used for in-process control of all coupling reactions. After assembly of the complete sequence the peptidyl resin was dried under vacuum overnight to give a weight of 8.86 g indicating a coupling efficiency of 55%. The peptidyl resin was stirred in a mixture of TFA/EDT/H_2_O/TIS (90%/5%/4%/1%, 10 mL/g) for 3 h to cleave the linear peptide from the resin. After the evaporation of the TFA, cold di-isopropyl ether was added to precipitate 5.53 g of crude linear peptide. The crude peptide was dissolved in water at a concentration of 3 mg/mL. The pH of the solution was adjusted between 7 and 8 with 0.1 M ammonium carbonate solution and the oxidation to the disulfide was allowed to proceed overnight to completion. The crude cyclized peptide (5.5 g) was purified on a 5 cm/24.2 cm column packed with 100A-10 µm-C18 using 0.1%TFA in acetonitrile/water with a gradient 18.5–28.5 in 30 min to yield 503.44 mg of THR-123 with a purity of 95.74% by HPLC.

### 2.2 Rat model of acute myocardial infarction and treatment protocol

Adult male Sprague-Dawley (SD) rats weighing 300 ± 20 g, obtained from JVC, Charles River Labs, were used in this study. The study was approved by the IACUC Committee of the U Mass Medical School, Worcester, MA and conducted in accordance with the guidelines from the National Institutes of Health for the care and use of Laboratory animals. All animals received humane care, and all efforts were made to minimize animal suffering. Three days prior to the week of assigned surgery, a blood sample was collected from the tail vein of each rat (pre-bleed). On the day of surgery, each rat was administered THR-123 or PBS intravenously 2 h prior to surgery. Surgery was performed sequentially after intubation and ventilation with 0.5% isoflurane anesthetic. Lead II ECG was monitored throughout the surgery. A thoracotomy was performed, and the main left coronary artery was occluded for 20 min using a snare technique, with few exceptions. Successful occlusion of the artery was confirmed by ST elevation in lead II ECG within the first 5 min of ligation, and by frequent ventricular arrhythmia after 10 min of ligation. The snare was released to restore blood flow. The original suture loop was left in place for subsequent re-occlusion on day 7. After the initiation of reperfusion, the chest cavity was closed with 2 layers of suture. Rats were weaned from the ventilator and observed until fully conscious and mobile. Antibiotic daily and analgesic every 8 h were provided. Additional serum samples were collected at 6, 24, 72 and 120 h post occlusion. THR-123, BMP-7 or vehicle treatments were delivered by intravenous injection at 24, 72 and 120 h post occlusion. On day 7, each rat was anesthetized (ketamine-xylazine) and a blood sample was taken from the inferior vena cava. The chest was opened, and the coronary artery was re-occluded as before. Immediately upon occlusion, Evans blue dye was slowly injected into the apex of the heart to stain the perfused (non-occluded) region. The heart was then arrested, excised and stored at −20 °C for 1.5 h. Ten 2 mm sections were made from the apex of the heart (slice 1) to the ligation area (slice 10). The living tissue in each slice was stained red by incubation in triphenyl-tetrazolium chloride (TTC). Each section was ultimately fixed in formalin and processed to paraffin block. TTC stained sections were photographed digitally for morphometric analysis (Spot 4.0 image analysis). For each slice, the following areas were identified and quantified: the perfused area, which was stained blue with Evans Blue, the ischemic area, which was stained red with TTC, and a pale area of presumed necrosis. The terminology included total “area at risk” (AR; stained red with TTC and unstained by Evans blue), the total “area of necrosis” (AN) defined as weakly-stained area within the area at risk (AR) and the total left ventricle area (LV). The AR and AN were expressed as a percentage of the left ventricle (LV). Similarly, the AN was expressed as a percentage of the cumulative AR. The average AR/LV, AN/LV, and AN/AR were then calculated for each group. Histopathology was performed on selected slices. Slices were stained by H&E and Trichrome, and processed to identify alpha-actin by immunohistochemistry.

The data reported herein was generated from a total of twenty-eight unmanipulated rats, sorted into two large groups by initial body weight (>300 g pre-surgery). Fourteen rats underwent surgery in each experiment. We used stratified randomization based on body weights of animals.

Each animal was individually weighed to get baseline body weights. The animals were then grouped into blocks based on their body weights. Within each weight block, animals were randomly assigned to the different control and treatment groups. This is to ensure a comparable average body weights across groups.

We carried out the animal-model in two sub-experiments in succession. In sub-experiment, the control group, BMP-7 group and THR-123 group had 5, 3 or 4, and 5 animals, respectively.

This allowed the effective handling of animals, facilitating ease of surgery and treatment of animals in each sub-experiment. We pooled the data from both the sub-experiments in a group-wise manner. Our observation (see results) that 84 percent infarct size reduction in THR-123 treatment group was significantly different (*p* = 0.008) from that of control (vehicle treated animals). Additional statistical analysis (Cohen's d) of data was carried out for robustness.

A common scoring system (0 to 4 point scale) is a method used for grading the severity of gross pathological changes in the pericardium in research/animal models. The specific scale system for inflammatory cell infiltration and fibrosis in heart tissue is typically defined as follows and has been used in our study.

The grading is as follows:

- 0 = No inflammation: The pericardium appears normal, shiny, glistening, and smooth.
- 1 = Slight pericarditis: Minor changes, likely early or very focal signs of inflammation.
- 2 = Moderate pericarditis: Clear signs of inflammation, such as a rough, granular texture due to fibrin deposition.
- 3 = Severe pericarditis: Pronounced inflammation and pathological changes, potentially including significant cellular infiltration and early signs of fibrous adhesions.
- 4 = Complete pericarditis: Extensive inflammation leading to significant tissue changes, likely including widespread fibrous adhesions or obliteration of the pericardial cavity (fibrosis).

We strictly followed blinding methods for surgery, treatment, and outcome evaluation including blinding the pathologists to the treatment groups during pericarditis scoring.

#### 2.2.1 Cell culture

Neonatal rat cardiac myocytes (Cell Applications) were cultured at a cell density of 2×10^5^/mL in six-well dishes. After being cultured with Medium 199 with 10% fetal calf serum (FCS) for 24 h, the medium was changed to Medium 199 with 1% FCS and incubated for 24 h. Thereafter, cardiac myocytes were cultured without serum for another 12 h. The cells were then stimulated with THR-123 or BMP-7. Cell lysates were prepared and analyzed for AKT phosphorylation and for Caspase-3 by ELISA. In these *in-vitro* experiments, replicates are both biological and technical. Replicates are from biological triplicates, and each biological sample was analyzed in triplicate (technical). Treatment of cells with BMP-7 or without served as positive or negative controls, respectively. Compound THR-123 is safe in cell cultures and did not cause cytotoxicity in our studies (20) and others (22, 23).

#### 2.2.2 AKT phosphorylation assay (ELISA)

The assay was performed using Cell Signaling's FastScan™ Phospho-Akt (Ser473) kit (# 80895) as per manufacturer's instructions. Validation, specificity and accuracy of ELISA have been well documented in the manufacturer's manual. In brief, the procedure includes incubation of the samples with a capture antibody, then with a detection antibody linked to HRP, forming a sandwich with phospho-Akt (Ser473). After washing the wells, add TMB substrate, and the signal intensity is proportional to the amount of phospho-Akt (Ser473).

#### 2.2.3 IL-6 (ELISA)

We tested THR-123 in a second cellular model of myocardial injury involving cellular damage caused by inflammatory cytokines, particularly TNF-α. To mimic the effects of TNF-α, we treated Neonatal rat cardiomyocytes (CM) with lipopolysaccharide (LPS, 100 ng/mL) for 12 h, followed by treatment with BMP-7 (143 nM) or the BMP mimetic (THR-123) (4 to 500 µM) for 24 h. Culture supernatants were assayed for pro-inflammatory cytokine release (IL-6) using Quantikine Rat IL-6 ELISA kit (# R6000B) from R&D Systems, as per manufacturer's instructions. In this *in-vitro* experiment, replicates are both biological and technical. Replicates are from biological triplicates, and each biological sample was analyzed in triplicate (technical)**.**

#### 2.2.4 Caspase-3 (ELISA)

Cell homogenates were assayed using rat Caspase-3 ELISA kit (Colorimetric) # NBP2-75024 from Novus Biologicals, as per manufacturer's instructions.

We have employed widely used well validated specific ELISA kits. Validation, specificity and accuracy of ELISA have been well documented in the manufacturer's manual.

Compound THR-123 is a small peptide and when tested at higher concentrations up to 500 µM, caused no cytotoxicity in our (20, 21) and other (22, 23) studies. Appropriate controls (without compound) and a positive/reference control (BMP-7) were maintained in cell culture experiments.

#### 2.2.5 Statistical analysis

All values were described as mean ± S.D. A Two-way ANOVA test was utilized to compare the statistical differences among groups. A *p*-value < 0.05 was considered statistically significant. Also, we did additional statistical analysis and included those results along with the relevant figures.

## 3 Results

### 3.1 Efficacy of THR-123 in the treatment of myocardial injury following ischemia (*in vivo*)

We evaluated THR-123 for its efficacy to alleviate myocardial injury caused by ischemia and reperfusion in the rat model of coronary artery ligation (see Figure 1). THR-123, BMP-7 or vehicle treatments were delivered by intravenous injection at 2 h before occlusion and at 24, 72 and 120 h post occlusion. Three days prior to the week of assigned surgery, a blood sample was collected from the tail vein of each rat (pre-bleed). Also, blood samples were collected at 6, 24, 72 and 120 h post occlusion, and on necropsy day 7.

The LAD occluded hearts from PBS or compound treated animals were cut into 10 cross-sectional slices (2 mm/slice) and each section was numbered and incubated separately in triphenyl-tetrazolium chloride (1% TTC in PBS) for 10 min at 37° C prior to fixing in 4% formaldehyde. Subsequently, each slice was photographed. All vehicle (PBS) treated animals showed significant myocardial infarction (Injured muscle in yellow). Treatment with THR-123 markedly reduced infarction indicating repair of Myocardial Ischemia.

**Figure 2:**
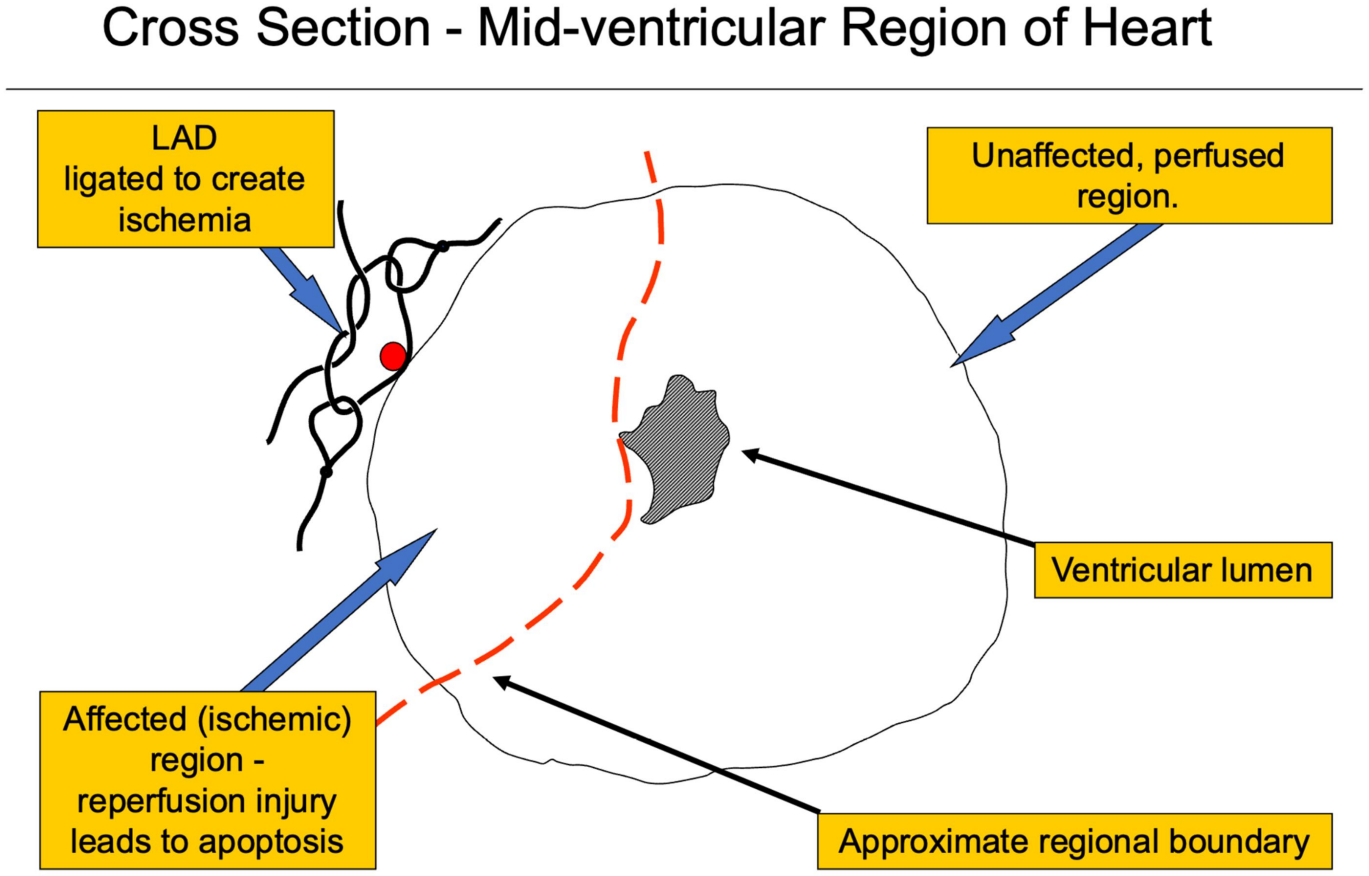
Cross sectional slice of heart. Unaffected, perfused region stained Evans blue dye prior to sacrifice.

**Figure 3:**
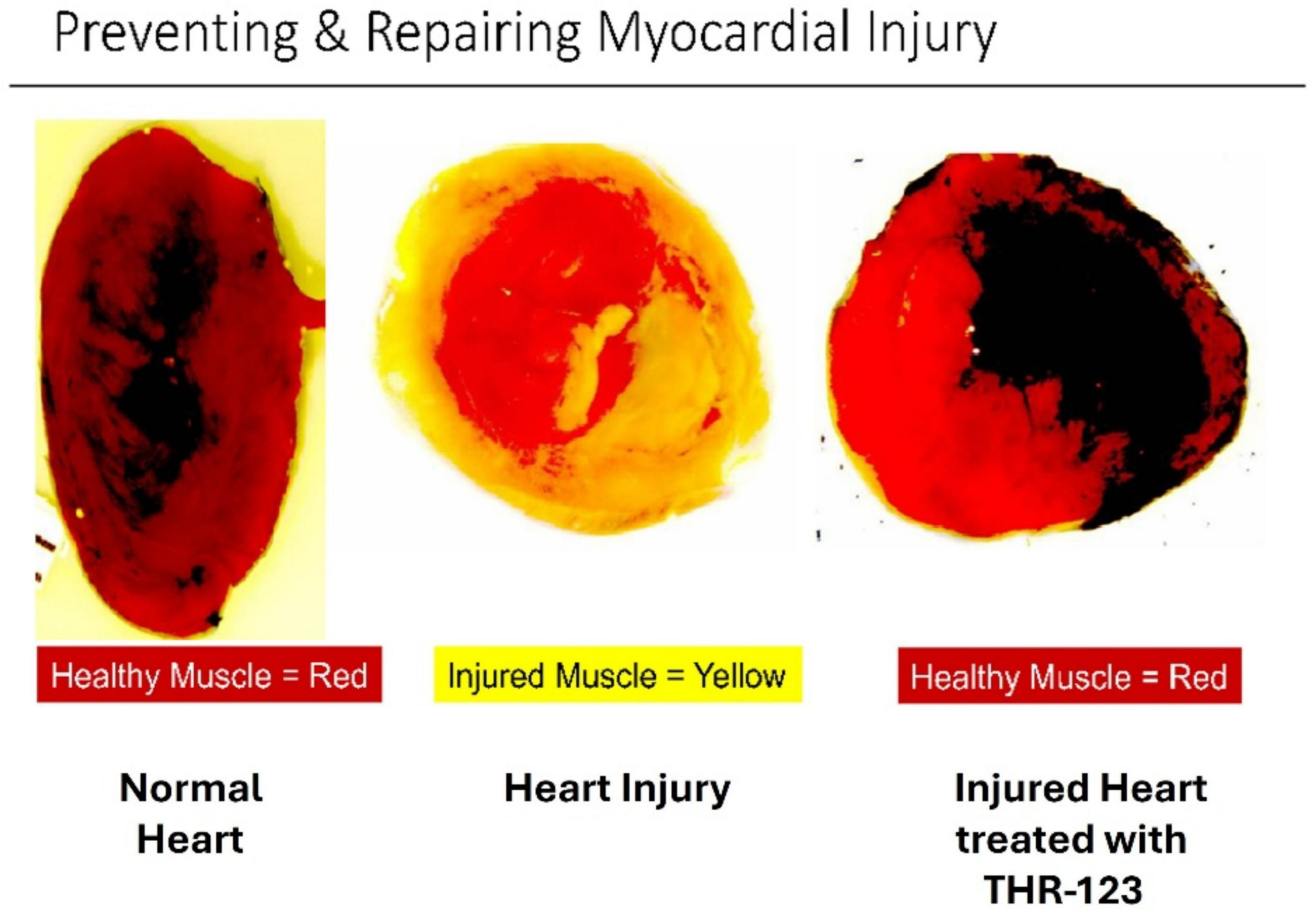
Preventing and repairing myocardial ischemic injury by THR-123.

On Day 7, each rat was anesthetized (ketamine-xylazine), the chest cavity was re-opened, and a terminal blood sample (1 mL) was withdrawn from the abdominal vein. Gross pathology (i.e., inflammation) was noted and graded on a 4 - point inflammation scale: 0 = no inflammation, 1 = slight, 2 = moderate, 3 = severe and 4 = complete pericarditis. The left coronary artery was then re-occluded using the existing stitch and a concentrated solution of Evans Blue dye in saline (20 mg/mL; 0.5 mL/rat) was injected into the heart's left anterior descending artery (LAD) to stain the non-ischemic cardiac tissue blue. The heart was then arrested by injection of a saturated solution of potassium chloride into the right ventricle. The heart was then excised, wrapped in cellophane and stored at −20° C. for 1.5 h. The cold heart was cut into 10 cross-sectional slices (2 mm/slice) (Figure 2) and each section was numbered and incubated separately in triphenyl-tetrazolium chloride (1% TTC in PBS) for 10 min at 37° C, prior to fixing in 4% formaldehyde. Subsequently, each slice was photographed (Figure 3). For each slice, the “area at risk (AR)” and the “area occupied by necrosis (AN)” were measured by morphometry. The ratio of AN to AR or ratio of volume of necrosis (VN) to volume at risk (VR) is a measure of the degree of damage resulting from the occlusion.

Control rats treated with PBS showed more severe pericarditis at necropsy as compared to minor pericarditis in rats treated with either compound (THR-123 or BMP-7). Infarction size reduction in animal models is commonly expressed either as a percentage of the entire left ventricle (LV) or, more accurately, as a percentage of the area/region at risk (AR). This is the standard method used in most scientific publications (24–26) to compare the efficacy of different treatments, and accordingly, we did (Table 1). Cohen's d values and interpretation for the group comparisons are shown in Table 2.

**Table 1:** Area of infarct (AI) or area of necrosis (AN) mm^2^ (See Figure 4).

Group	Area of Necrosis (AN) mm^2^ Mean ± S.D.	Ratio: Area of AN/ Area of Risk (AR) Mean ± S.D.	% relative to control	% reduction in Infarct
Control (Vehicle)	12.625 ± 7.46	0.048 ± 0.024*	100	
THR-123	2.256 ± 3.79	0.0076 ± 0.0125*	15.8	84.2
BMP-7	11.78 ± 9.72	0.0416 ± 0.039	86.45	13.55

* *P*=0.008(significantly different).

*P* > 0.5(Not significantly different).

**Table 2:** Cohen's d values & interpretation.

Figure#	Control(vehicle) Vs THR-123		Control(vehicle) Vs BMP-7	
	Cohen's d	Effect	Cohen's d	Effect
Figure 4	2.05	Large	0.22	Small
Figure 5	2.25	Large	0.51	Medium
Figure 6	2.05	Large	0.22	Small
Figure 7	5.30	Large	4.35	Large

Interpretation: Cohen’s d values, 0.2 (Small Effect) 0.5 (Medium Effect) 0.8 & above (Large Effect).

**Figure 4:**
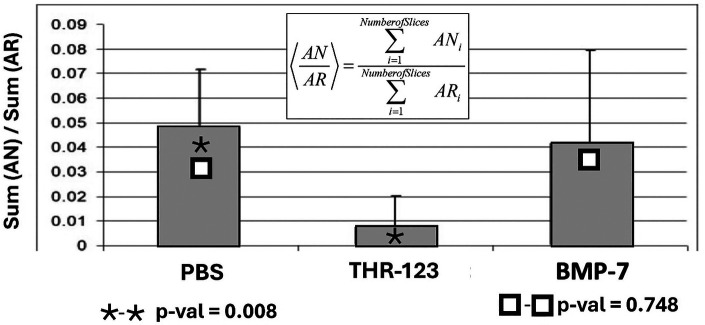
THR-123 treated animals showed marked decline in myocardial infarction size compared to PBS treated animals. Animals underwent necropsy on post-surgical day 7. Each occlusion was confirmed by ECG analysis during the surgery and ultimately after Evans blue injection on Day 7 at necropsy. All but one rat demonstrated successful occlusion by Evans Blue at necropsy. Animals PBS-treated, BMP-7 treated or THR-123 treated were evaluated at necropsy. Areas of infarct were clearly identified in all heart samples and morphometry was performed on all the harvested slices. Treatment-related differences in gross pathology and morphometry were observed. Results are expressed as Ratio of Area of Infarct (AI) to Area of Risk (AR) (Mean ± SD) see Table 1. *Vehicle (PBS) vs. BMP mimetic (THR-123), *P* = 0.008. △ Vehicle (PBS) vs. BMP-7, *P* = 0.748. Cohen's d values & interpretation for the group comparisons are shown in Table 2.

Previously we have extensively tested our compound THR-123 for dose-response, dose regimen and bioavailability in number of animal models (20), and the doses selected for THR-123 and BMP-7 in this study were based on their activity profiles. THR-123 markedly decreased myocardial infarct size (Table 1, Figure 4) and pericardial inflammation. The animals treated with 10 mg/kg animal body weight of the compound THR-123 showed an 84% reduction in unweighted AN/AR ratio as compared to PBS-treated animals (Table 1) whereas animals treated with 160 μg/kg animal body weight recombinant BMP-7 had only a 14% reduction in unweighted AN/AR ratio as compared to PBS-treated animals (Table 1, Figure 4). Similarly, the compound THR-123 showed an 84% reduction in VN/VR ratio as compared to PBS-treated animals (Figure 6). Furthermore, animals treated with THR-123 showed a 78% reduction in inflammation score as compared to PBS-treated animals (Figure 7).

**Figure 5:**
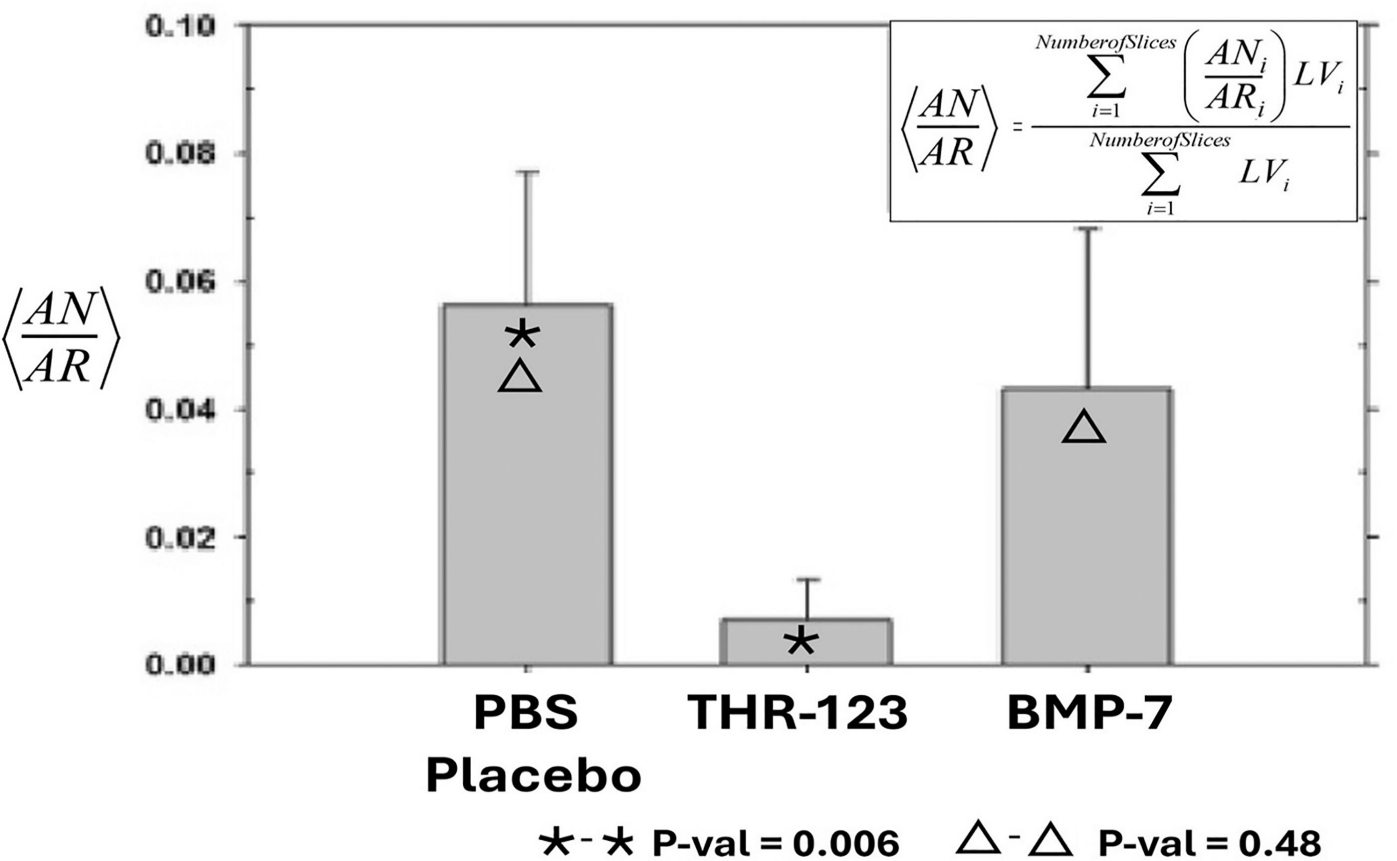
THR-123 (BMP mimetic) reduced the left ventricular area weighted ratio AN/AR [the area occupied by necrosis (AN) to area at risk (AR)] by 84% compared to vehicle (PBS), in the LAD occlusion model of myocardial infarction. Animals PBS-treated, BMP-7 treated or BMP mimetic (THR-123) -treated were evaluated at necropsy. Areas of infarct were clearly identified in all heart samples and morphometry was performed on all the harvested slices. Results are expressed as Ratio of Area of Necrosis (AN) to Area of Risk (AR) (Mean ± SD). *Vehicle (PBS) vs. BMP mimetic (THR-123), *P* = 0.006. △ Vehicle (PBS) vs. BMP-7, *P* = 0.48. Cohen’s d values & interpretation for the group comparisons are shown in Table 2.

**Figure 6:**
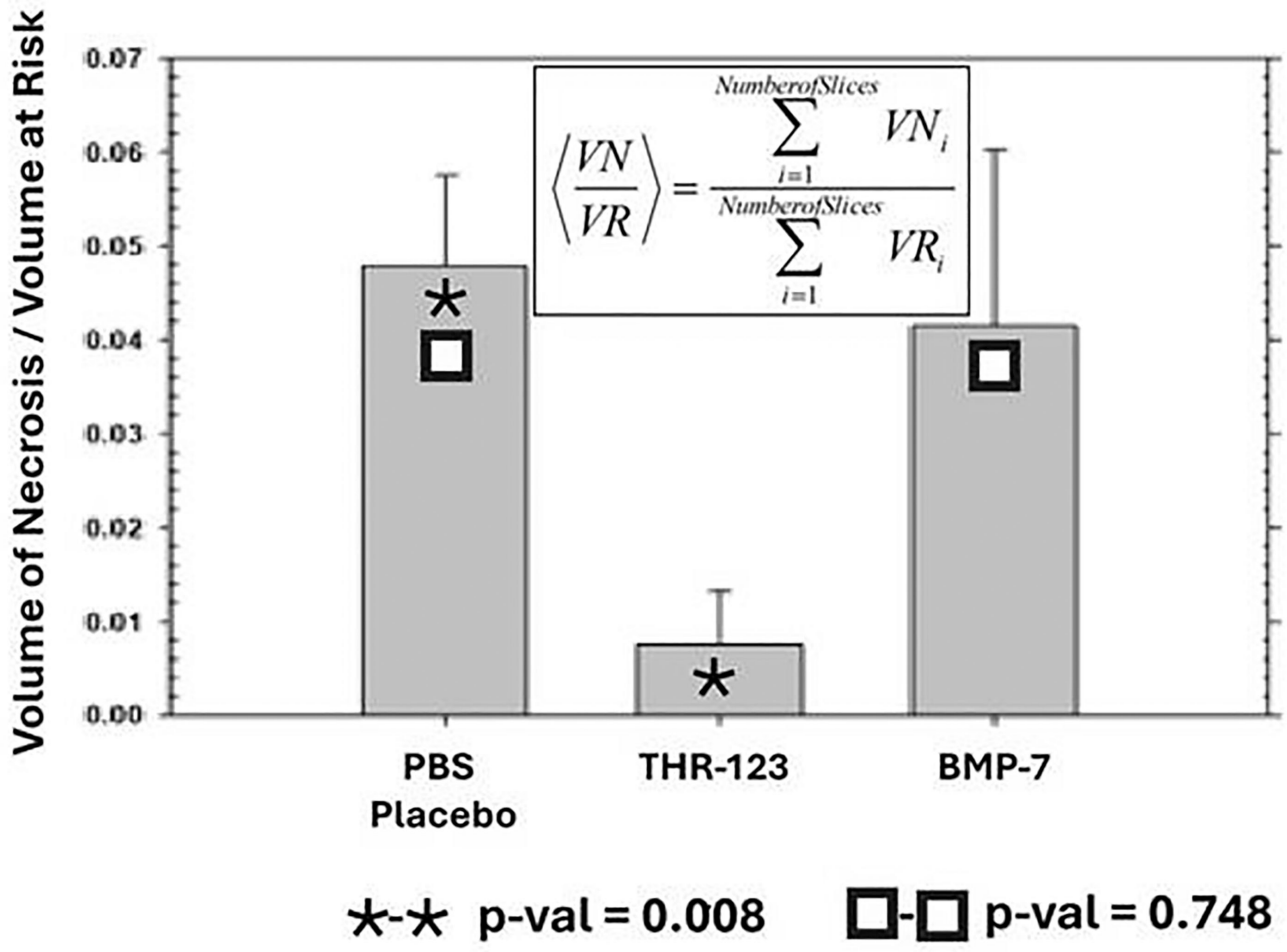
THR-123 (BMP mimetic) reduced VN/VR ratio [volume of necrosis (VN) to volume at risk (VR)] in the LAD occlusion model of myocardial infarction. Animals PBS-treated, BMP-7 treated or BMP mimetic (THR-123) -treated were evaluated at necropsy. Areas of Infarct were clearly identified in all heart samples and morphometry was performed on all the harvested slices. Results are expressed as ratio of Volume of Necrosis (VN) to Volume at Risk (VR) (Mean ± SD). *Vehicle (PBS) vs. BMP mimetic (THR-123), *P* = 0.008**.** △ Vehicle (PBS) vs. BMP-7, *P* = 0.748**.** Cohen’s d values & interpretation for the group comparisons are shown in Table 2.

**Figure 7:**
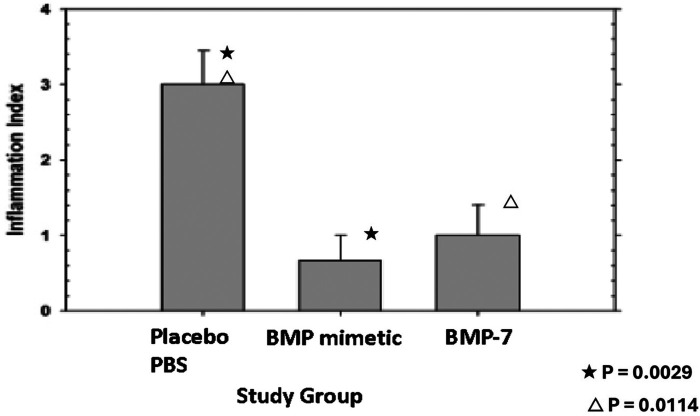
THR-123 (BMP mimetic) reduced the degree of cardiac inflammation in the LAD occlusion model of myocardial infarction. Animals PBS-treated, BMP-7 treated or BMP mimetic (THR-123) -treated were evaluated for inflammation. * Vehicle (PBS) vs. BMP mimetic (THR-123), *P* = 0.0029. △ Vehicle (PBS) vs. BMP-7, *P* = 0.0114. Cohen’s d values & interpretation for the group comparisons are shown in Table 2.

These results thus explain the important cardioprotective effects of THR-123 and may provide a novel pharmacological intervention in myocardial injury.

### 3.2 THR-123 is cardio protective in myocardial injury in *in vitro* models

To determine the mechanism of action of THR-123 on cardiomyocytes, we examined its *in vitro* effects in three different cellular models: Chemotherapy-induced cardiomyocyte injury (Apoptosis Model), Endotoxin-mediated cardiomyocyte injury (Inflammation Model), and serum deprivation of cardiomyocytes (Ischemia Model).

*In vitro* models of cardiomyocytes commonly use indirect stimuli such as lipopolysaccharide (LPS) for inflammation and serum deprivation for ischemia. The regulation of bone morphogenetic protein (BMP) signaling pathways plays a critical role in these models influencing cell survival, apoptosis, and the inflammatory response. LPS, a component of Gram-negative bacteria, is a potent activator of the Toll-like receptor 4 (TLR4) signaling pathway, which triggers a cascade of inflammatory responses, including the activation of NF-*κ*B and the production of pro-inflammatory cytokines like TNF-α and IL-6. This effectively mimics the inflammatory environment seen in conditions like post-myocardial infarction injury (27, 28). There is significant crosstalk between the LPS-activated inflammatory pathways (like NF-*κ*B and p38 MAPK) and BMP signaling (29). Serum deprivation contributes to nutrient deficiency and general stress. BMP has been shown to promote the survival of serum-deprived cardiomyocytes and inhibit apoptosis by enhancing the expression of anti-apoptotic molecules like Bcl-x(L). BMP-7 also exhibits cardioprotective effects, acting as an anti-fibrotic factor and promoting cardiomyocyte proliferation in regenerating hearts.

In summary, these *in vitro* models are valuable tools for dissecting the molecular mechanisms of cardiac injury and repair, allowing researchers to study direct drug effects.

#### 3.2.1 THR-123 is anti-apoptotic in chemotherapeutic myocardial injury model (*in vitro*)

**Figure 8:**
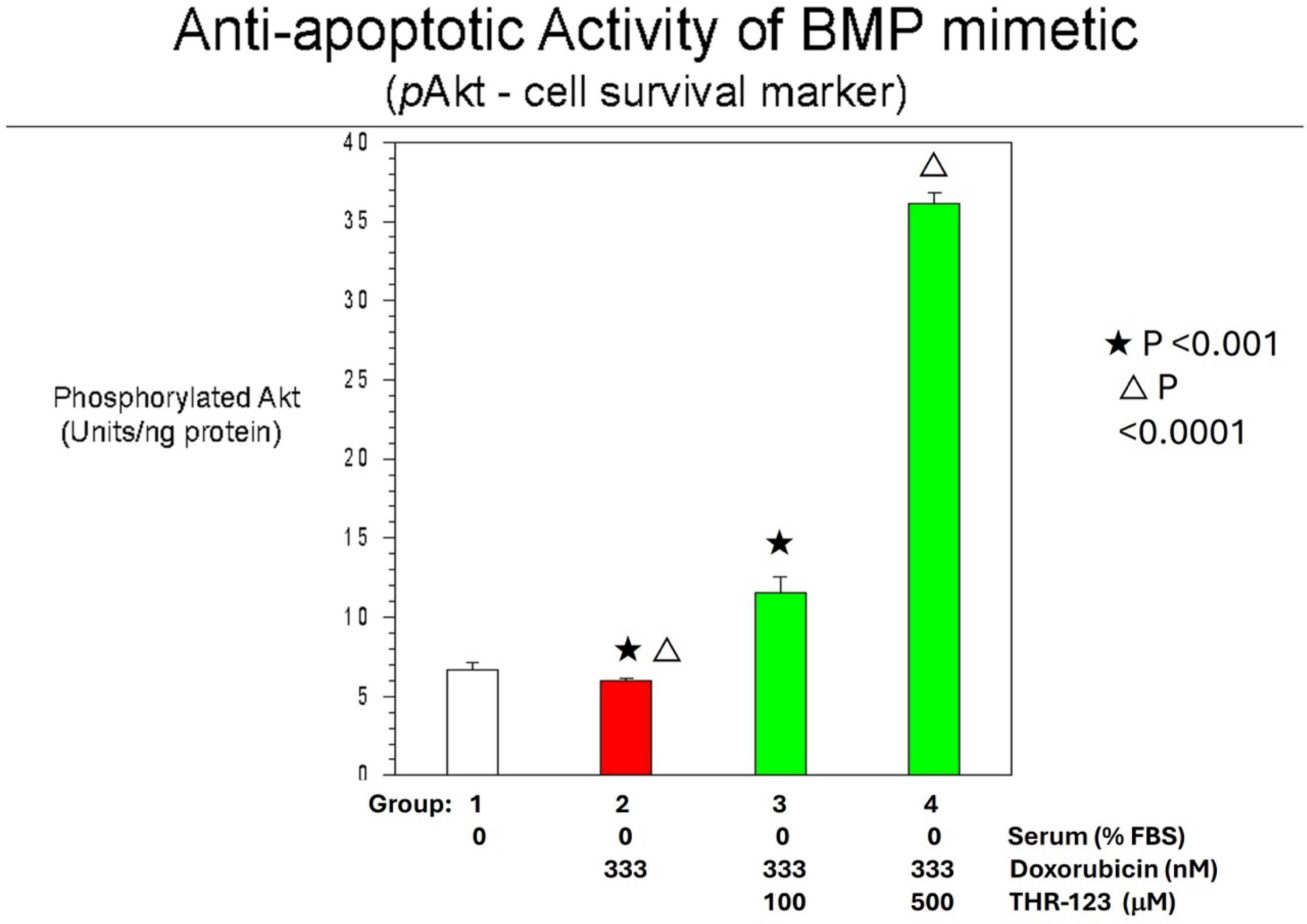
THR-123 (BMP mimetic) significantly increased cellular Akt phosphorylation compared to doxorubicin (Adriamycin) alone. Cell lysates were prepared & analyzed for AKT phosphorylation by ELISA. *Doxorubicin alone vs. Doxorubicin + BMP mimetic (THR-123, 100 µM), *P* < 0.001. △ Doxorubicin alone vs. Doxorubicin + BMP mimetic (THR-123, 500 µM), *P* < 0.001.

**Figure 9:**
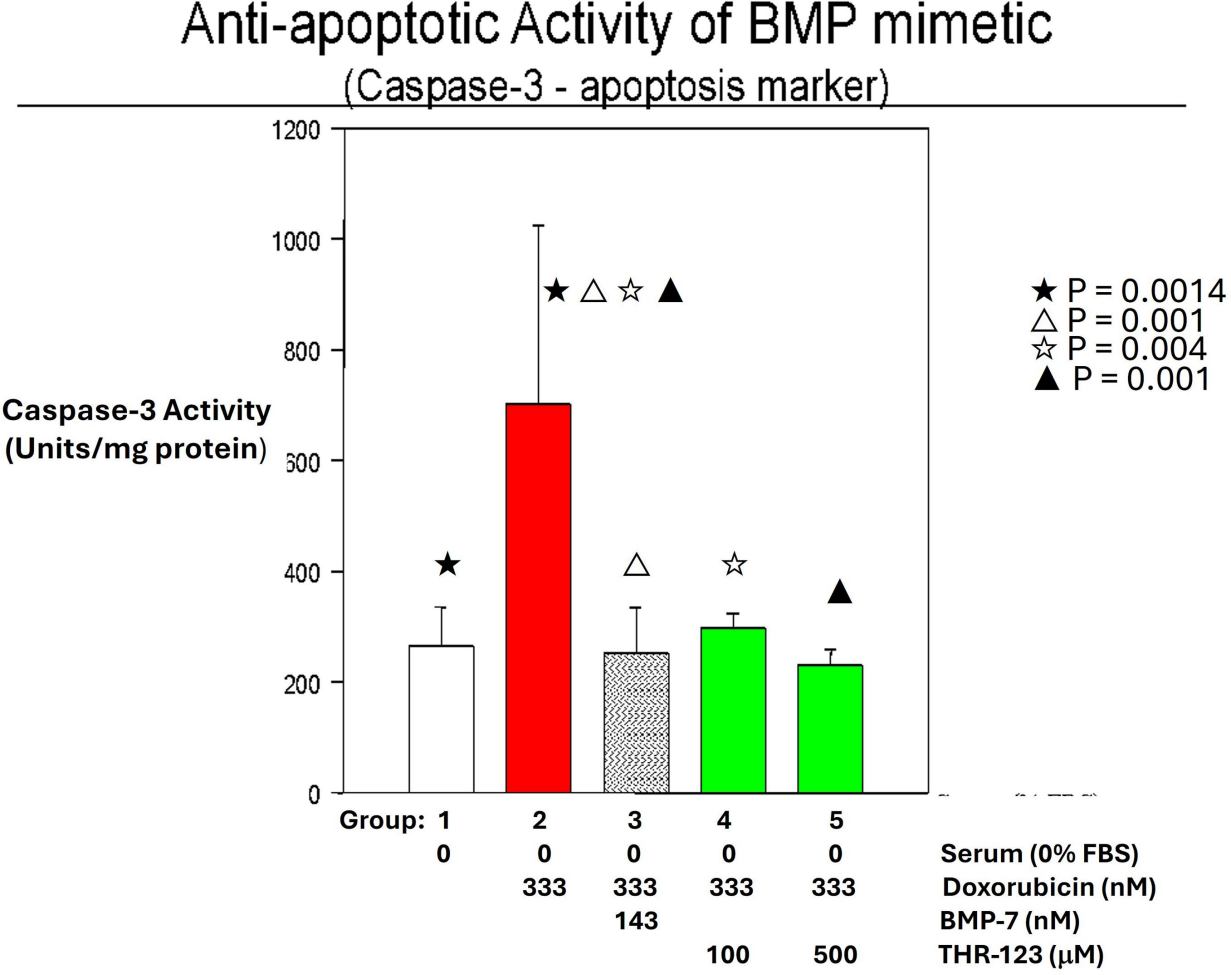
THR-123 (BMP mimetic) effectively inhibited doxorubicin (Adriamycin) induced caspase-3 activity (apoptosis marker) in cardiomyocytes. Cell lysates were prepared & analyzed for Caspase-3 by ELISA. ★Cardiomyocytes were cultured in medium without serum vs. medium containing Doxorubicin, *P* = 0.0014. **△** Doxorubicin vs. BMP-7, *P*= 0.001. ⋆ Doxorubicin vs. BMP mimetic (THR-123 100 µM), *P*=0.004. ▴ Doxorubicin vs. BMP mimetic (THR-123 500 µM), *P*= 0.001.

First, we used a chemotherapeutic drug-induced myocardial injury model, in which we examined the effect of THR-123 on cellular Akt phosphorylation (phospho-Ser473), which is known to be involved in promoting cardiomyocyte survival, function and contractility, and also on Caspase-3 activity, a cellular enzyme responsible for apoptosis (30). Neonatal rat cardiomyocytes (CM) were pre-treated with 0.33 µM Adriamycin (Doxorubicin) for 24 h, followed by treatment with BMP-7 (143 nM) or THR-123 (100 and 500 µM) for 60 h. Our test compound significantly increased cellular Akt phosphorylation (*p*< 0.001) compared to Adriamycin alone (Figure 8), and inhibited Adriamycin induced Caspase-3 activity (*p* = 0.004) (Figure 9).

The analysis of data in Figure 8 for Tukey HSD (honesty significant difference) & interpretation are shown in Table 3.

**Table 3:** Tukey HSD (honest significant difference) analysis and interpretation.

Groups compared	Difference between Group means	HSD value	Groups’ difference: is higher or lower than HSD?	Tukey’s test interpretation: Is difference significant? Yes or No
Group 1 vs. 2	0.5	0.8757	Lower	No
Group 1 vs. 3	5.0	0.8757	Higher	Yes
Group 1 vs. 4	29.5	0.8757	Higher	Yes
Group 2 vs. 3	5.5	0.8757	Higher	Yes
Group 3 vs. 4	24.5	0.8757	Higher	Yes

The analysis of data in Figure 9 for Tukey HSD (honesty significant difference) & interpretation are shown in Table 4.

**Table 4:** Tukey HSD (honest significant difference) analysis and interpretation.

Groups compared	Difference between Group means	HSD value	Groups’ difference: is higher or lower than HSD?	Tukey’s test interpretation: Is difference significant? Yes or No
Group 1 vs. 2	430	204.4	Higher	Yes
Group 2 vs. 3	450	204.4	Higher	Yes
Group 2 vs. 4	370	204.4	Higher	Yes
Group 2 vs. 5	460	204.4	Higher	Yes
Group 3 vs. 4	80	204.4	Lower	No
Group 4 vs. 5	90	204.4	Lower	No

#### 3.2.2 THR-123 is anti-inflammatory in cardiomyocyte injury model (*in vitro*)

**Figure 10:**
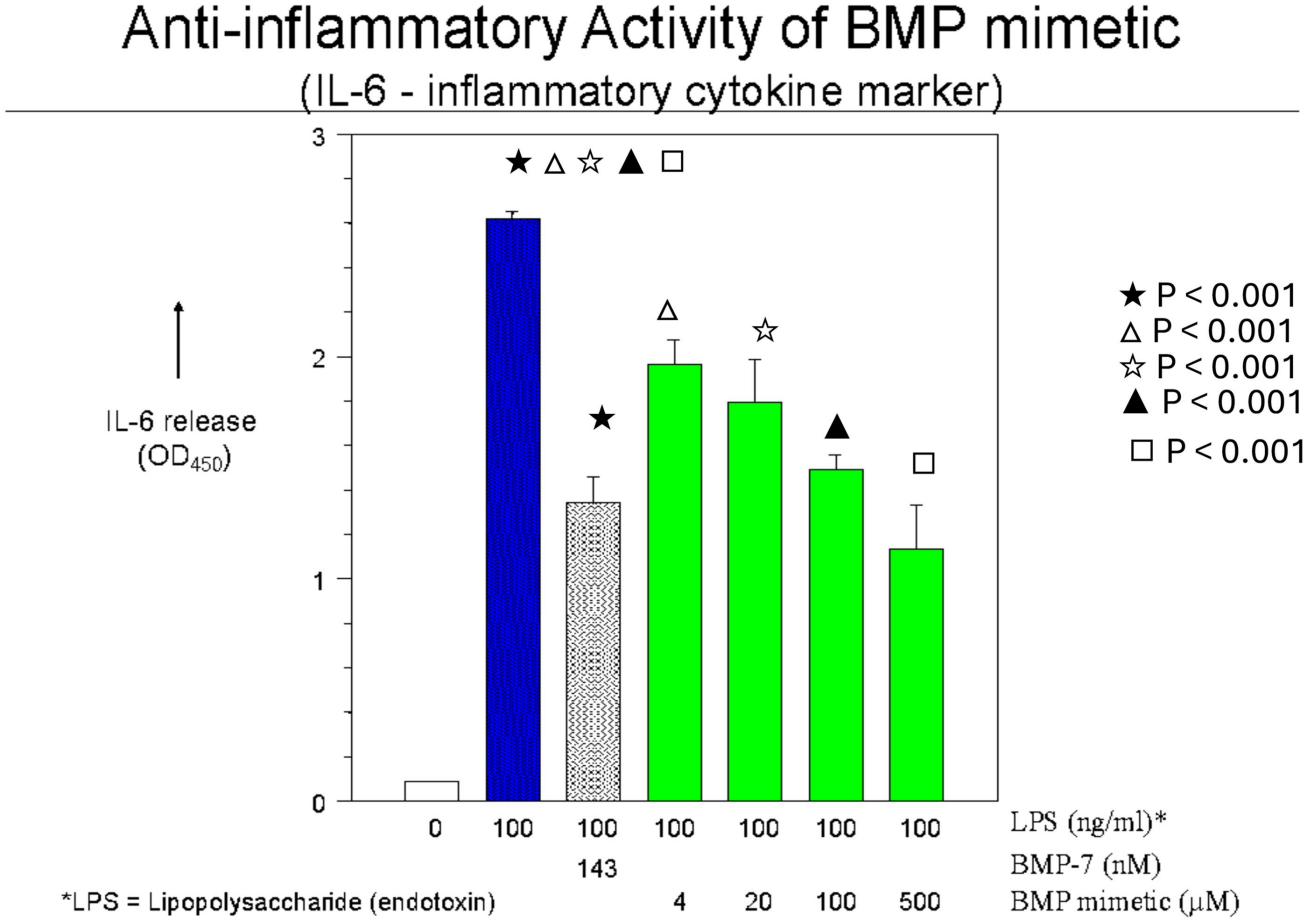
THR-123 (BMP mimetic) effectively inhibited LPS (lipopolysaccharide) induced inflammation (pro- inflammatory cytokine IL-6 release) in cardiomyocytes. Cardiomyocytes were treated with lipopoly-saccharide (LPS, 100 ng/mL) for 12 h, followed by treatment with BMP-7 (143 nM) or BMP mimetic (4 to 500 µM) for 24 h., as indicated in ‘Methods’. Culture supernatants were assayed for pro-inflammatory cytokine release (IL-6) using ELISA. ★ LPS vs. LPS + BMP-7, *P* < 0.001. **△** LPS vs. LPS + BMP-7 mimetic (THR-123, 4 µM), *P* < 0.001. ⋆ LPS vs. LPS + BMP-7 mimetic (THR-123, 20 µM), *P* < 0.001. ▴LPS vs. LPS + BMP-7 mimetic (THR-123, 100 µM), *P*< 0.001. ◻ LPS vs. LPS + BMP-7 mimetic (THR-123, 500 µM), *P* < 0.001.

**Figure 11:**
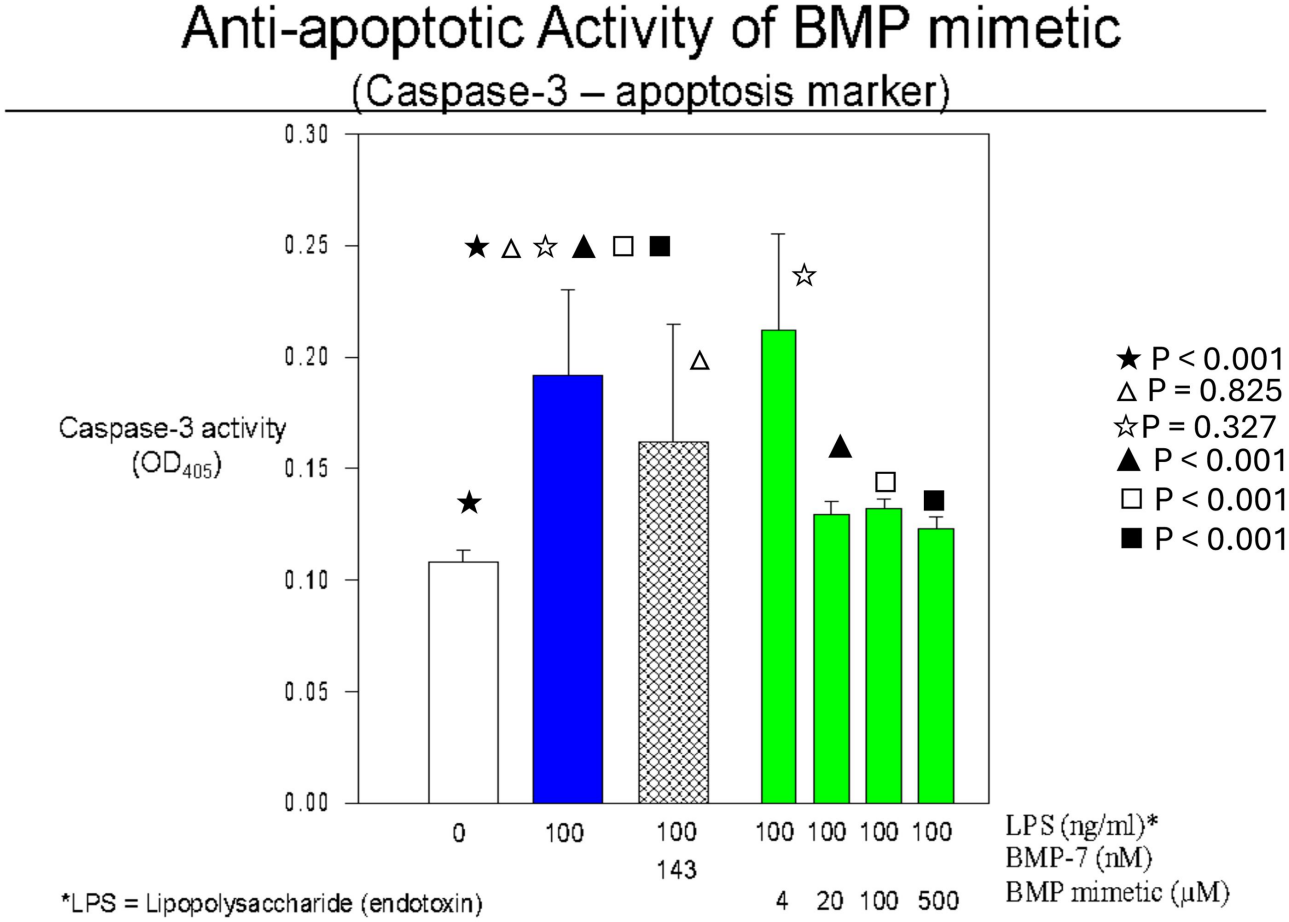
THR-123 (BMP mimetic) effectively inhibited LPS (lipopolysaccharide) induced caspase-3 activity (apoptosis marker) in cardiomyocytes. Cell lysates were prepared & analyzed for Caspase-3 by ELISA. ★ Medium alone vs. LPS, *P* < 0.001. △ LPS vs. LPS + BMP-7, *P* = 0.825. ⋆ LPS vs. LPS + BMP-7 mimetic (THR-123, 4 µM, *P* = 0.327. ▴ LPS vs. LPS + BMP-7 mimetic (THR-123, 20 µM, *P* < 0.001. **◻** ◻°LPS vs. LPS + BMP-7 mimetic (THR-123, 100 µM, *P* < 0.001. ◼ LPS vs. LPS + BMP-7 mimetic (THR-123, 500 µM, *P* < 0.001.

We then tested THR-123 in a second cellular model of myocardial injury involving cellular damage caused by inflammatory cytokines particularly TNF-α. To mimic the effects of TNF-α, we treated neonatal rat cardiomyocytes (CM) with lipopolysaccharide (LPS, 100 ng/mL) for 12 h, followed by treatment with BMP-7 (143 nM) or THR-123 (4 to 500 µM) for 24 h. Our test compound effectively inhibited LPS induced pro-inflammatory cytokine release (IL-6) (Figure 10) as well as Caspase-3 activity (Figure 11).

The analysis of data in Figure 10 for Tukey HSD (honesty significant difference) & interpretation are shown in Table 5.

**Table 5:** Tukey HSD (honest significant difference) analysis and interpretation.

Groups compared	Difference between Group means	HSD value	Groups’ difference: is higher or lower than HSD?	Tukey’s test interpretation: Is difference significant? Yes or No
Group 2 vs. 3	1.275	0.0465	Higher	Yes
Group 2 vs. 4	0.675	0.0465	Higher	Yes
Group 2 vs. 5	0.835	0.0465	Higher	Yes
Group 2 vs. 6	1.125	0.0465	Higher	Yes
Group 2 vs. 7	1.485	0.0465	Higher	Yes
Group 4 vs. 5	0.158	0.0465	Higher	Yes
Group 5 vs. 6	0292	0.0465	Higher	Yes
Group 6 vs. 7	0.36	0.0465	Higher	Yes

The analysis of data in Figure 11 for Tukey HSD (honesty significant difference) & interpretation are shown in Table 6.

**Table 6:** Tukey HSD (honesty significant difference) analysis and interpretation are shown below.

Groups compared	Difference between Group means	HSD value	Groups’ difference: Is higher or lower than HSD?	Tukey’s test interpretation: Is difference significant? Yes or No
Group 1 vs. 2	0.0784	0.05806	Higher	Yes
Group 2 vs. 3	0.0275	0.05806	Lower	No
Group 2 vs. 4	0.02	0.05806	Lower	No
Group 2 vs. 5	0.06	0.05806	Higher	Yes
Group 2 vs. 6	0.059	0.05806	Higher	Yes
Group 2 vs. 7	0.065	0.05806	Higher	Yes
Group 4 vs. 5	0.08	0.05806	Higher	Yes
Group 5 vs. 6	0.001	0.05806	Lower	No
Group 6 vs. 7	0.006	0.05806	Lower	No

#### 3.2.3 THR-123 is cardioprotective in ischemic injury model (*in vitro*)

**Figure 12:**
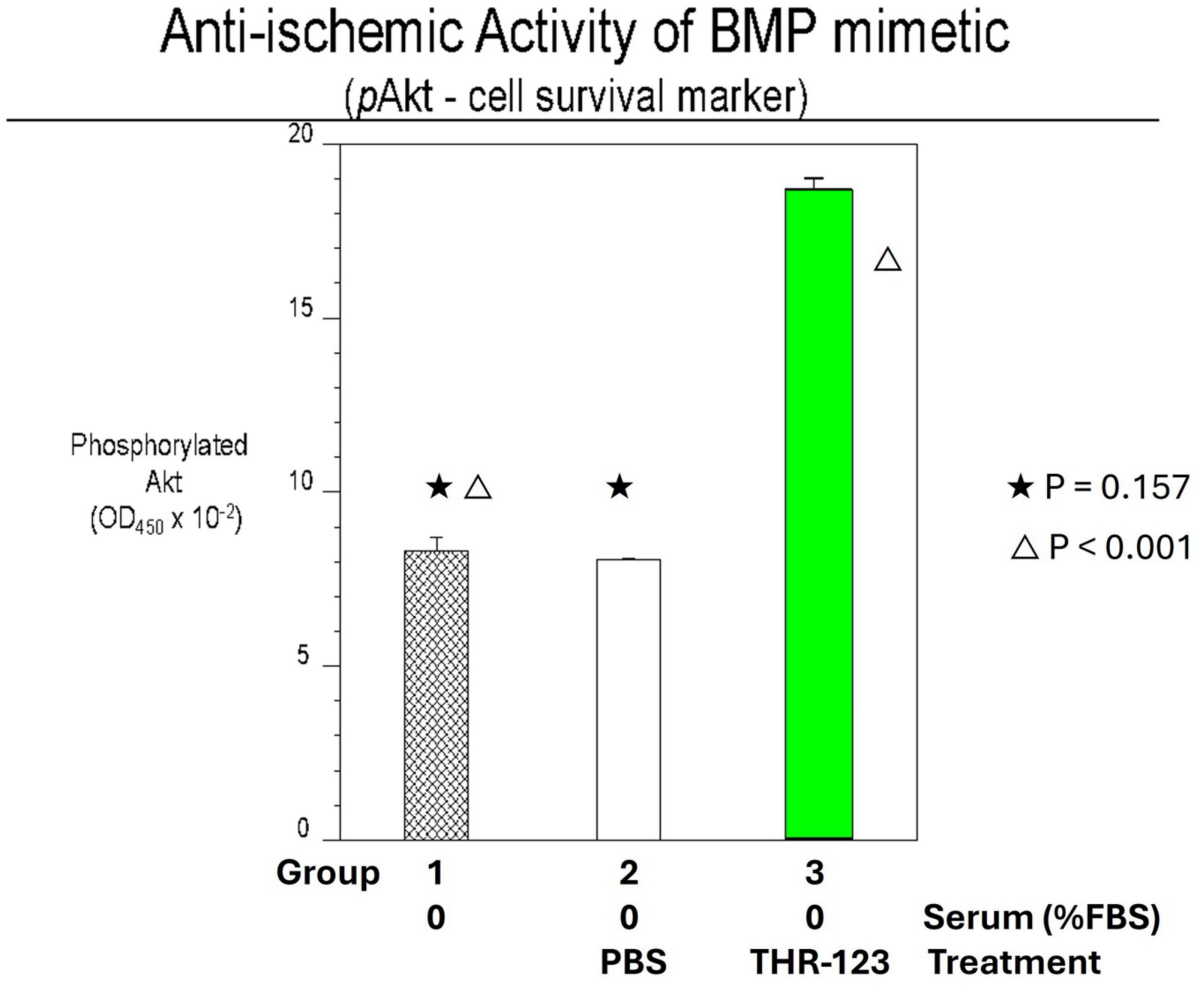
THR-123 significantly increased Akt Ser473 phosphorylation (*p* < 0.001) in cardiomyocytes subjected to ischemic injury. Cell lysates were prepared & analyzed for AKT phosphorylation by ELISA. ★ Serum-free medium vs. Medium + PBS, *P* = 0.157. **△** Serum-free Medium vs. Medium + BMP mimetic (THR-123, 100 µM), *P* < 0.001.

Finally, in a cellular model of ischemic injury (by serum deprivation), primary neonatal rat cardiomyocytes were cultured in medium with 10% growth supplement (Cell Applications) for 24 h at 37° C., 5% CO2. Cells were starved of growth supplement for 12 h, Controls received medium alone. Cells were incubated with culture medium alone, BMP- 7 (143 nM) or THR-123 (100 µM) for 60 h to detect Akt phosphorylation at Serine 473 (Cell Signaling Technology). Our compound significantly increased Akt Ser473 phosphorylation (*p* <0.001) (Figure 12) and protected cardiomyocytes from apoptosis.

The analysis of data in Figure 12 for Tukey HSD (honesty significant difference) & interpretation are shown in Table 7.

**Table 7:** Tukey HSD (honest significant difference) analysis and interpretation.

Compared	Difference Between Group means	HSD value	Groups’ difference: Is higher or lower than HSD?	Tukey’s test interpretation: Is difference significant? Yes Or No
Group 1 vs. 2	0.2	0.3563	Lower	No
Group 1 vs. 3	10.5	0.3563	Higher	Yes
Group 2 vs. 3	10.7	0.3563	Higher	Yes

These results thus explain the important cardioprotective effects of our THR-123 compound and may provide a novel pharmacological intervention in myocardial injury.

## 4 Discussion

Here we have shown, for the first time, that in a rat model of acute myocardial infarction, an i.v. administration of a novel BMP-7 mimetic (THR-123) reduced myocardial infarct size and alleviated IRI. THR-123 is a small peptide agonist of BMP-7 signaling pathway, designed from the crystal structure of BMP-7 (19).

Acute myocardial infarction (MI) continues to be the most common cause of HF (heart failure) despite the tremendous advancements in the treatment of MI over the past 20 years (31). The cellular events involved in repair of the infarcted heart can be divided into three distinct, but overlapping phases: the inflammatory phase, the proliferative phase and the maturation phase (5, 32). During the inflammatory phase, release of alarmins by necrotic and apoptotic cardiomyocytes activates innate immune pathways, leading to recruitment of leukocytes in the infarcted myocardium (33). Clearance of dead cells and matrix debris from the infarct by professional phagocytes activates anti-inflammatory cascades leading to suppression of the inflammatory response and transition to the proliferative phase of infarct healing (34). During the proliferative phase, activated myofibroblasts deposit large amounts of extracellular matrix proteins in the infarcted area, while activation of angiogenesis ensures perfusion of the highly cellular and metabolically active wound. The maturation phase follows, as activation of anti-fibrotic pathways limits the fibrogenic response, leading to formation of a mature scar that contains a small amount of cross-linked collagenous matrix.

A widely used animal model is the Coronary artery Ligation model where the left anterior descending coronary artery (LAD) is ligated to induce ischemia in the heart. In this animal model of myocardial infarction, various growth factors like vascular endothelial growth factor (VEGF) (35), basic fibroblast growth factor (bFGF) (36), (TGF-*β*) (37), platelet-derived growth factor (PDGF) (38), and hepatocyte growth factor (HGF) (39) have shown potential to alleviate the damage by promoting angiogenesis, cell survival, and tissue repair within the infarcted area. While these growth factors are promising, the translation to clinical practice for myocardial infarction treatment is still under investigation due to concerns about potential adverse effects like tumor growth or uncontrolled angiogenesis.

Various other strategies have been devised to reduce the clinical consequences of myocardial infarction, especially prevention of cardiomyocyte cell death. Activation of signaling pathways is a particularly interesting option to improve the functional performance and survival rate of cardiomyocytes. Of interest is the bone morphogenetic proteins (BMPs)/signaling, which have some potentially beneficial therapeutic effects on cardiac development and regulate cellular events associated with cardiac repair, by modulating injurious, inflammatory, apoptotic, and fibrogenic responses. In a mouse model of acute myocardial infarction, an i.v. injection of BMP-2 reduced infarct size in mice when given after left anterior descending artery ligation. Mice treated with BMP-2 are characterized by a reduced rate of apoptotic cardiomyocytes both in the border zone of the infarcts and in the remote myocardium (40). The data suggest that BMP-2 treatment may have considerable therapeutic potential in individuals with acute and chronic myocardial ischemia by improving the contractility of cardiomyocytes and preventing cardiomyocyte cell death. In another study (41), the survival rate of BMP-7-treated AMI group was significantly improved post two weeks of LAD ligation, compared to the saline group. Moreover, the role of the TGF-*β*1 signaling pathway in BMP-7-mediated cardioprotective effects was investigated by analyzing the expression levels of TGF-*β*1, Smad-2 and Smad-3 in the infarct zone, border zone, and non-infarct zone. Results indicated that BMP-7 attenuated myocardial fibrosis through counteracting TGF-*β*1 signaling pathway, thereby exerting cardioprotective effects. The ability of BMPs to alleviate ischemia and reperfusion injury suggests that they could be a potential therapeutic target for treating heart conditions like myocardial infarction (MI). However, any potential therapeutic benefit of restoring BMP-7 functions using systemic rhBMP-7 is hampered by bioavailability, induction of ectopic bone formation, induction of neutralizing autoantibodies against BMPs, and a range of potential adverse effects (18, 21).

The important step in developing a BMP-7 mimetic is first and foremost that the compound activates the BMP pathways while separating out the osteogenic activity that has been shown to lead to the formation of ectopic bone when treating soft tissue injury. Furthermore, the whole BMP-7 protein is subject to inhibition by natural inhibitory proteins such as follistatin, chordin and gremlin (42). A mimetic would likely be immune to this inhibition. Small peptide mimetics are also less likely than biologics to induce an immune response. Peptide mimetics of 20 residues or less can be synthesized chemically, which is a much simpler, cheaper, and reliable form of manufacturing than fermentation, the primary method for producing biologics.

### 4.1 THR-123 structure & activity

**Figure 13:**
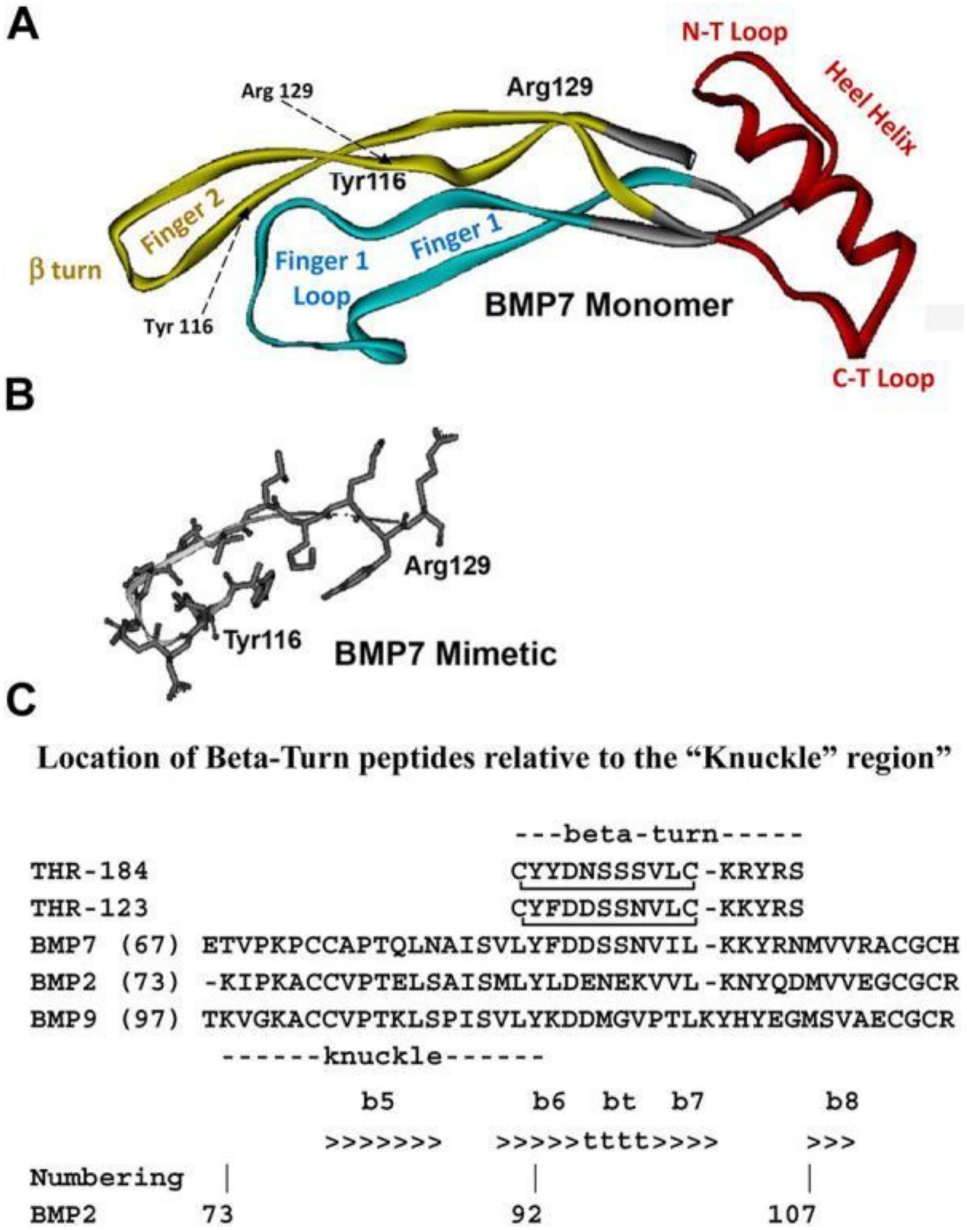
Structure diagrams of the BMP-7 monomer and the region covered by the mimetic. **(A)** A ribbon diagram showing the secondary structure of the BMP monomer, which contains three structural regions: antiparallel beta sheets of “Finger 1” (with the large terminal loop), “Finger 2” (with the tight beta-turn), and the “Heel” alpha helix. Initial targets for mimetic development were the terminal loops of fingers 1 and 2, loops at the C-terminal, and N-terminal loops at the ends of the Heel helix. **(B)** The region around the beta turn of Finger two that proved to have activity similar to BMP-7 and became the lead for further mimetic development. **(C)** The “beta-turn” region covered by the mimetic is immediately C-terminal to the “knuckle” region covered by other BMP mimetics. Residue position numbers are based on BMP-2 residue numbers. Secondary structure: beta sheet (>>>>), segments of which are labelled e.g., “b6”; beta turn (bt, tttt). Peptide disulfide bond: C_C.

As explained in Carlson et al. (21), we designed BMP-7 mimetic peptide THR-123 from the x-ray structure of BMP-7 (19, 20). The method identifies solvent accessible regions likely involved in receptor binding, then identified and optimized the best leads based on *in vitro* assays. These peptides, which are anti-inflammatory, anti-apoptotic and anti-fibrotic, cover the tight beta-turn region of finger 2 (Figure 13A). The fact that they display no osteogenic activity and do not bind to BMPR-IB (ALK6) correlates with the fact that osteogenic peptides identified to date (43–48) cover the physically separate “knuckle” region (Figure 13C), and provides a physical explanation for the specificity of activity (21).

THR-123 selectively binds the BMP type I receptors ActR-IA (ALK2) and BMPR-IA (ALK3) and type II receptor BMPR-II. It does not bind BMPR-IB (ALK6) (7, 20). THR-123 had no therapeutic activity in ALK3 knock-out (deleted) mice (20). It exhibits robust anti-inflammatory, anti-apoptotic and antifibrotic and regenerative effects in several experimental models of acute and chronic kidney diseases (20, 21).

In the present study, we employed a coronary artery Ligation model in the rat to determine the efficacy of THR-123 in ameliorating the effects of myocardial ischemia. Animals that survived ligation of the coronary artery underwent necropsy on post-surgical day 7. Areas of infarct were clearly identified in all heart samples and morphometry was performed on all the harvested heart slices. Treatment-related differences in gross pathology and morphometry were noteworthy. Control rats treated with PBS showed more severe pericarditis at necropsy as compared to minor pericarditis in rats treated with BMP-7 or THR-123. THR-123 markedly decreased myocardial infarct size and pericardial inflammation. The animals treated with THR-123 at the dose of 10 mg/kg animal body weight, showed an 84% reduction in AN/AR ratio as compared to PBS-treated animals, whereas animals treated with recombinant BMP-7 at a dose of 160 µg/kg animal body weight had only a 14% reduction in AN/AR ratio as compared to PBS-treated animals. Similarly, THR-123 showed an 84% reduction in VN/VR ratio as compared to PBS-treated animals. Furthermore, animals treated with THR-123 showed a 78% reduction in inflammation score as compared to PBS treated animals. Although BMP-7 was very effective reducing inflammation, it probably requires a higher dose to better reduce myocardial infract size. These results demonstrate the ability of THR-123 to alleviate ischemia and reperfusion injury, suggesting that it could be a potential therapeutic target for treating heart conditions like myocardial infarction.

The mechanism of action of BMP-7 in cardiomyocytes has been well investigated. The studies indicated that BMP-7 promotes cardiomyocyte regeneration in adult mice, potentially offering a promising strategy for Cardiomyocyte protection, proliferation and regeneration after injury. BMP-7 promotes cardiomyocyte proliferation through the activation of BMPR1A/ACVR1 and ACVR2A/BMPR2 receptors, leading to downstream signaling involving Smad-5, ERK, and AKT (49). In the absence of BMP signaling, cardiomyocytes become arrested in the S-phase of the cell cycle, which prevents progression to mitosis and results in heart regeneration failure. Interestingly, BMP signaling can also rescue neonatal mouse cardiomyocytes and human fibroblasts from hydroxyurea-induced replication stress (50).

Previously, we have shown that ALK3 modulates THR-123 actions by ALK3 knockout studies (20), and this was further demonstrated in recent studies by Salido-Medina et al. (22) in cardiomyocytes. More recently, Alverez-Cubela et al. (23) presented evidence to confirm that ALK3 mediates THR-123 actions. However, in these studies and in the present study, target inhibitors like Dorsomorphin remain to be tested.

Both Smad signaling (51, 52) and AKT phosphorylation (53, 54) are important for cardiac protection and rescuing from Myocardial injury. In fact, the activation of canonical signaling pathway (Smad-1/5/(8)9 phosphorylation) in response to THR-123 in cardiomyocytes has been well investigated and previously published (22). THR-123 rescued the expression of BMPR-IA (ALK3) and associated pSmad-1/5/(8)9 signaling in the left ventricle (LV). The increased phosphorylation of Smad-1/5/(8)9 in the left ventricle in response to THR-123, rescued ventricular hypertrophy and disfunction in mouse models of cardiac remodeling and fibrosis (22).

### 4.2 AKT phosphorylation

Research confirms that BMP uses non-canonical pathways, predominantly AKT pathway. The AKT pathway, also known as the PI3K/Akt pathway, is a crucial intracellular signaling pathway involved in regulating cell growth, survival, and metabolism. It plays a significant role in heart health and disease, including myocardial infarction (MI). Specifically, the AKT pathway is implicated in mitigating the damage caused by myocardial ischemia-reperfusion injury (MIRI), a common complication of MI.

In the context of myocardial infarction, the AKT pathway plays a protective role by:

- Inhibiting cardiomyocyte apoptosis**:** AKT activation can prevent programmed cell death (apoptosis) of heart muscle cells (cardiomyocytes) following ischemia (lack of blood flow) and reperfusion (restoration of blood flow).
- Reducing infarct size: By promoting cell survival and inhibiting apoptosis, the AKT pathway can limit the extent of tissue damage caused by MI.
- Restoring cardiac function: Activation of the AKT pathway may help restore the heart's ability to pump blood effectively after an MI.
- Modulating inflammation and oxidative stress**:** The AKT pathway can influence the inflammatory response and oxidative stress, both of which are involved in MIRI.

#### 4.2.1 Key roles of BMP-induced AKT activation

BMP-7 triggers AKT phosphorylation after heart attack (MI) to promote cardiomyocyte survival, proliferation, and cardiac repair, reducing scar size by activating pro-survival pathways (like PI3K/Akt), enhancing mitochondrial function, and influencing inflammation, ultimately leading to a better heart function. AKT, once activated by BMPs, promotes cell cycle reentry, inhibits cell death, and helps coordinate healing, contrasting with excessive fibrosis often seen in BMP deficiency.

#### 4.2.2 Crosstalk between PI3/AKT phosphorylation and smad signaling

Following BMP-7 treatment, a significant increase in *p*-Smad-1/5/(8)9 and *p*-PI3K expression, resulting in downstream activation of *p*-Akt and *p*-mTOR, was observed (55). AKT phosphorylation crosstalks with and can regulate Smad 1/5/8 signaling (42), playing a crucial, generally protective role in rescuing the heart from Myocardial Infarction by influencing cell survival, proliferation, and fibrosis, through their interplay with the Smad1/5/8 (BMP pathway), often promoting survival/angiogenesis, and Akt broadly enhanced recovery (56).

In summary, BMPs act as critical growth factors after a heart attack using the AKT pathway as a central hub to promote new heart muscle cell formation and survival alongside improving the heart's energy use and modulating the damaging fibrotic response leading to infarct reduction and better long-term function.

Thus, the PI3K/Akt pathway is a major intracellular signaling pathway that regulates cell survival, growth, and proliferation of cardiomyocytes (5). Mechanistically, PI3K phosphorylates phosphatidylinositol-4,5-bisphosphate (PIP2) to generate phosphatidylinositol-3,4,5 trisphosphate (PIP3), which then recruits Akt, a serine/threonine kinase. Once activated, Akt phosphorylates various downstream targets, including mTOR, which promotes cardiomyocyte cell survival and growth. Caspases are key mediators of programmed cell death (apoptosis). Among them, Caspase-3, a frequently activated death protease, catalyzes the specific cleavage of many key cellular proteins (33).

In the present study, to determine the mechanism of action of the BMP mimetic THR-123, we examined the *in vitro* effects of our compound in three different cellular models.

### 4.3 THR-123 is anti-apoptotic in a myocardial injury model (*in vitro*)

It is known that the PI3K/Akt pathway participates in the process of myocardial injury induced by Doxorubicin (Adriamycin), a chemotherapeutic agent. In the present study, we used a Doxorubicin-induced myocardial injury model, in which we examined the effect of THR-123 on cellular Akt phosphorylation (phospho-Ser473), which is known to involve in promoting cardiomyocyte survival, function and contractility, as well as on caspase-3 activity, a cellular enzyme responsible for apoptosis. When neonatal rat cardiomyocytes (CM) were pre-treated with doxorubicin followed by treatment with THR-123, our test compound (THR-123) promoted cardiomyocyte survival by significantly increasing cellular Akt phosphorylation and prevented the loss of cardiomyocytes by inhibiting Adriamycin-induced apoptosis (caspase-3 activity).

### 4.4 THR-123 is anti-inflammatory in endotoxin-mediated cardiomyocyte injury model (*in vitro*)

The phosphoinositide 3-kinase (PI3 K)/Akt signaling pathway has been shown to play an important role in negatively regulating LPS-induced acute inflammatory responses *in vitro* and *in vivo* (34, 57–63). Inhibition of PI3K/Akt signaling enhances LPS-induced activation of NF-*κ*B, AP-1, and Egr-1 transcription factors and gene expression of TNF-α and tissue factor in cultured human monocytic cells (34). Similar effects were observed *in vivo*, where inhibition of PI3K/Akt enhanced LPS-induced coagulation and inflammation in endotoxemic mice (63). Recent studies have shown that Akt dampens LPS-induced NF-*κ*B activation by phosphorylating and, hence, inactivating glycogen synthase kinase (GSK) 3*β*, which negatively regulates its downstream target, NF-*κ*B, and potently suppresses LPS-induced proinflammatory responses and endotoxic shock (34, 63). It has been shown that Bone morphogenetic protein (BMP), specifically BMP-7, attenuates lipopolysaccharide (LPS)-induced inflammatory responses in cardiac cells by activating the phosphoinositide 3-kinase (PI3 K)/Akt signaling pathway, which plays a crucial role in maintaining anti-inflammatory environments (64). Further studies have shown that the Smad-PI3K-Akt-mTOR pathway specifically inhibits pro-inflammatory cytokine secretion (TNF-α, IL-6 and MCP-1), enhances anti-inflammatory cytokines (IL-10 and IL-1ra) and plays a key role in M2 macrophage polarization (31, 65). These studies strongly suggest that the PI3K/Akt pathway limits inflammation in endotoxin mediated cardiac injury.

We tested our compound in a cellular model of myocardial injury involving cellular damage caused by inflammatory cytokines, particularly TNF-α. To mimic the effects of TNF-α, we treated neonatal rat cardiomyocytes (CM) with lipopolysaccharide (LPS), followed by treatment with the BMP mimetic THR-123. Our compound effectively inhibited LPS induced pro-inflammatory cytokine release (IL-6) as well as apoptosis (Caspase-3 activity). The fact that both pericarditis scores and *in vitro* cytokine (IL-6) data are consistent indicates that THR-123 is anti-inflammatory. These results suggest that THR-123 is effective attenuating endotoxin-induced cardiomyocyte dysfunction and that the mechanisms involve the preserved activation of Akt phosphorylation, resulting in attenuation of LPS-induced expression of pro-inflammatory cytokine IL-6 release, and inhibition of apoptosis.

### 4.5 THR-123 is cardioprotective in ischemic injury model (by serum deprivation) (*in vitro*)

It has been shown that serum deprivation triggers apoptotic responses through mitochondrial pathways leading to mitochondrial dysfunction. The detection of mitochondrial permeability transition (MPT) events, specifically the opening of the mitochondrial permeability transition pore (mPTP), provides an early indication of the initiation of cellular apoptosis, including in cardiomyocytes, as MPT can lead to mitochondrial dysfunction and the release of pro-apoptotic factors (66, 67). BMP-2 and BMP-4 enhance the expression of anti-apoptotic markers Bcl-x1 (68) and Bcl-2 (69), respectively. Interestingly, BMP-2 promoted survival via its signaling Smad-1 and inhibited apoptosis of serum deprived myocytes (68). On the other hand, BMP-4 induced phosphorylation of Akt and the protective effect of BMP-4 on cell viability was significantly blocked by a PI3K/Akt inhibitor, indicating that the PI3K/Akt pathway is required for the anti-apoptotic effects of BMP-4. In the present study, we tested our compound in a cellular model of ischemic injury (by serum deprivation), using primary neonatal rat cardiomyocytes. THR-123 significantly increased Akt Ser473 phosphorylation (*p* <0.001) and protected cardiomyocytes from apoptosis.

These results thus explain the important cardioprotective effects and mechanism of action of THR-123 in alleviating myocardial injury.

Due to the limited regenerative capacity of the myocardium, infarcted areas heal through scar formation, and this often leads to heart remodeling, characterized by dilation, segmental hypertrophy of remaining tissue, and ultimately, cardiac failure and death (63). Merino et al. (8) have shown that bone morphogenetic protein through BMPRIA-mediated pSmad-1/5/(8)9 signaling protects the left ventricle (LV) against maladaptive remodeling and facilitates reverse ventricular remodeling and functional recovery in mice. Recently, in mice subjected to TAC, Salido-Medina et al. (22) established the cardioprotective effects of our BMP mimetics, THR-123 and THR-184. Daily i.p. injection with either peptide during four weeks, starting on the day of TAC surgery, (i) rescued the expression of BMPR-IA(ALK3) and associated pSmad-1/5/(8)9 signaling in the LV, (ii) prevented transcriptional activation of remodeling-associated genes (Col1a1, *β*-MHC, BNP and Acta-2), (iii) attenuated LV structural damage (hypertrophy and fibrosis), and (iv) diminished LV dysfunction (systolic and diastolic).Thus the growing evidence from our study and others indicate the beneficial effects of THR-123 in both acute and chronic cardiac injuries to restore heart functions.

Interestingly, the use of BMP mimetics for therapeutic applications has been gaining momentum in different clinical fields. Qadir et al. (70) have shown that THR-123, effectively induced nongenetic conversion of human pancreatic exocrine cells to insulin-expressing and functional—glucose-responsive—endocrine cells with a capacity for rapid reversal of diabetes *in vivo*. Recently, Alvarez-Cubela et al. (23) have shown that THR-123 administered i.p. without the administration of pluripotent cells reduces hypoglycemia in diabetic mice. This work independently demonstrates a safer and simpler alternative to genetic reprogramming or exogenous administration of stem cells as a treatment for diabetes. It is the first report of direct reprogramming using a BMP mimetic. More recently, Haipei et al. (71) have shown that THR-123 can activate the BMP pathway to inhibit Snail-1 and Epithelial Mesenchymal Transition (EMT) in retinal pigment epithelial cells and thereby regulate Proliferative Vitreoretinopathy (PVR) occurrence. THR-123 could provide a potential therapy for PVR. We evaluated THR-184, another BMP mimetic, in clinical studies of Acute Kidney injury (NIH, ClinicalTrials.gov Identifier: NCT01830920) and found it to be safe and well tolerated by patients. There was a noticeable reduction in the incidence of AKI in the patient subgroup with pre-existing CKD treated with the highest dose of THR-184 (21).

## 5 Conclusion

We have shown here, for the first time, that treatment with the BMP-7 mimetic, THR-123, protects cardiomyocytes, and limits infarct size after myocardial ischemia and reperfusion injury. Mechanistically, THR-123 activates Akt phosphorylation and inhibits inflammation and apoptosis in cardiomyocytes. These results support and extend the pivotal role of BMP/signaling in cardiac function. Recent studies have identified a new role for BMP/signaling as an endogenous inhibitor of cardiac apoptosis, inflammation and fibrosis, that maintains cardiac functions. However, noteworthy differences exist between BMP-7 and its Mimetic. Unlike other BMPs, a BMP-7 mimetic THR-123 does not bind ALK 6 (type 1 receptor), is not osteogenic and does not induce ectopic bone formation (21). Moreover, unlike BMP-7, THR-123 is orally bioavailable and has been shown to be effective in preventing renal injury in animal models, reversing fibrosis and restoring function (20). Importantly, the evidence from our present study and others (22) suggest that THR-123 could rescue the heart from both acute and chronic cardiac injuries. Developing therapies for treating myocardial infarction and preventing the ensuing cardiac dysfunction resulting in heart failure has been difficult and the translation of compounds with beneficial effects in animal models is fraught with difficulty.

Since we show here that blocking the deleterious effects of several types of injury with a growth factor mimetic THR-123 has the potential for development as a therapy for the treatment of myocardial injury that leads to cardiac failure. The administration of the novel BMP-7 mimetic, THR-123, holds promise as a strategy to repair and restore cardiac functions following cardiac injury.

## Data Availability

The raw data supporting the conclusions of this article will be made available by the authors, without undue reservation.
